# Poly[disodium [diaqua­tri-μ_2_-oxalato-dimagnesium(II)]]

**DOI:** 10.1107/S1600536808019508

**Published:** 2008-07-05

**Authors:** Xue-An Chen, Fang-Ping Song, Xin-An Chang, He-Gui Zang, Wei-Qiang Xiao

**Affiliations:** a College of Materials Science and Engineering, Beijing University of Technology, Ping Le Yuan 100, Beijing 100124, People’s Republic of China; b Institute of Microstructure and Properties of Advanced Materials, Beijing University of Technology, Ping Le Yuan 100, Beijing 100124, People’s Republic of China

## Abstract

The title compound, {Na_2_[Mg_2_(C_2_O_4_)_3_(H_2_O)_2_]}_*n*_, is isotypic with its Co analogue. There are two crystallographically independent oxalate groups in the asymmetric unit, one lying on an inversion center and the other on a general position. Mg^2+^ ions are ligated by H_2_O mol­ecules and bridged by tri- and tetra­dentate oxalate ligands, forming ladder-like double chains that are held together *via* O—H⋯O hydrogen bonds, with Na^+^ cations located between the chains to balance the charge.

## Related literature

For related literature, see: Audebrand *et al.* (2003 ▶); Brown & Altermatt (1985 ▶); Dean *et al.* (2004 ▶); Kolitsch (2004 ▶); Lethbridge *et al.* (2003 ▶); Lu *et al.* (2004 ▶); Miessen & Hoppe (1987 ▶); Price *et al.* (2000 ▶); Schefer & Grube (1995 ▶); Shannon (1976 ▶).
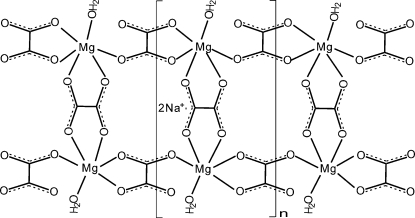


## Experimental

### 

#### Crystal data

Na_2_[Mg_2_(C_2_O_4_)_3_(H_2_O)_2_]
*M* *_r_* = 394.70
Monoclinic, 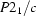

*a* = 5.8460 (12) Å
*b* = 15.726 (3) Å
*c* = 7.0190 (14) Å
β = 101.11 (3)°
*V* = 633.2 (2) Å^3^
*Z* = 2
Mo *K*α radiation
μ = 0.34 mm^−1^
*T* = 290 K
0.4 × 0.2 × 0.2 mm

#### Data collection

Rigaku AFC-7R diffractometer
Absorption correction: ψ scan (Kopfmann & Huber, 1968 ▶) *T*_min_ = 0.912, *T*_max_ = 0.943
2457 measured reflections
2280 independent reflections
2027 reflections with *I* > 2σ(*I*)
*R*_int_ = 0.029
3 standard reflections every 150 reflections intensity decay: 1.2%

#### Refinement

*R*[*F* ^2^ > 2σ(*F* ^2^)] = 0.033
*wR*(*F* ^2^) = 0.095
*S* = 1.11
2280 reflections
118 parameters
All H-atom parameters refined
Δρ_max_ = 0.54 e Å^−3^
Δρ_min_ = −0.56 e Å^−3^

### 

Data collection: *AFC Diffractometer Control Software* (Rigaku, 1994 ▶); cell refinement: *AFC Diffractometer Control Software*; data reduction: *AFC Diffractometer Control Software*; program(s) used to solve structure: *SHELXS97* (Sheldrick, 2008 ▶); program(s) used to refine structure: *SHELXL97* (Sheldrick, 2008 ▶); molecular graphics: *ATOMS* (Dowty, 1999 ▶); software used to prepare material for publication: *SHELXL97*.

## Supplementary Material

Crystal structure: contains datablocks global, I. DOI: 10.1107/S1600536808019508/bq2084sup1.cif

Structure factors: contains datablocks I. DOI: 10.1107/S1600536808019508/bq2084Isup2.hkl

Additional supplementary materials:  crystallographic information; 3D view; checkCIF report

## Figures and Tables

**Table 1:** Hydrogen-bond geometry (Å, °)

*D*—H⋯*A*	*D*—H	H⋯*A*	*D*⋯*A*	*D*—H⋯*A*
O7—H7a⋯O4^i^	0.78 (3)	2.06 (3)	2.8335 (13)	170 (2)
O7—H7b⋯O6^ii^	0.84 (3)	1.89 (3)	2.6952 (13)	162 (3)

Symmetry codes: (i) 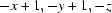; (ii) 
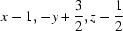
.

**Fig. 1.**
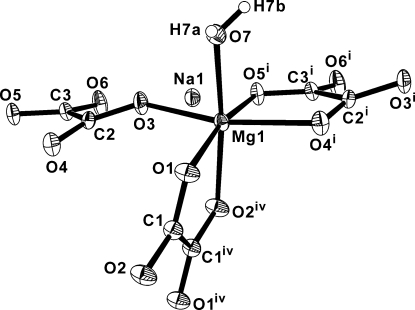
The connectivity in Na2Mg2(C2O4)3.2H2O, shown with displacement ellipsoids at the 50% probability level. [Symmetry codes: (i) -1 + x, y, z; (iv) 1 - x, 1 - y, 1 - z]

**Fig. 2.**
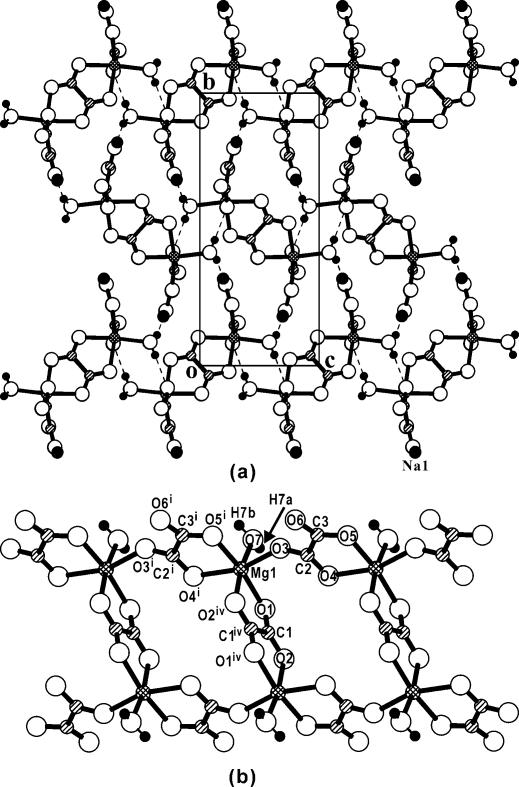
The crystal structure of Na2Mg2(C2O4)3.2H2O projected along the [100] direction (a) as well as the single chain of [Mg2(C2O4)3(H2O)2]n2n- (b). H···O hydrogen bond contacts are shown as dashed lines; symmetry codes are the same as those in Figure 1.

## supplementary crystallographic information

### Comment

Oxalates are of considerable interest because many of them are natural minerals and in addition, the oxalate anion can adopt different coordination modes to bind metals to form infinite chains, sheets and networks, leading to the rich structural chemistry (Lu *et al.*, 2004; Dean *et al.*, 2004; Audebrand *et al.*, 2003). In the system of mixed oxalates, A*_x_*B*_y_*(C_2_O_4_)*_z_*.nH_2_O, combining alkali-metal elements (A) and alkali-earth-metal cations (B), only one compound, Cs_2_Mg(C_2_O_4_)_2_.4H_2_O, has been previously found, and it has a layered structure character in which layers of MgO_4_(H_2_O)_2_ octahedra paralel to (10–1) are separated by corrugated layers of nine-coordinated Cs atoms (Kolitsch, 2004). During our exploratory syntheses of novel hydrated borate materials, we have obtained a new member of the A*_x_*B*_y_*(C_2_O_4_)*_z_*.nH_2_O family of compounds, Na_2_Mg_2_(C_2_O_4_)_3_.2H_2_O. It has a one-dimensional character consisting of [Mg_2_(C_2_O_4_)_3_(H_2_O)_2_]_n_^2n-^ infinite chains. We describe its synthesis and crystal structure here for the first time.

The title compound is isotypic with its Co analogue (Price *et al.*, 2000) and the crystal structure consists of Na^+^ and Mg^2+^ cations, [C_2_O_4_]^2-^ groups, and H_2_O molecules as the fundamental structural building units (Fig. 1). Mg^2+^ ions are ligated by H_2_O molecules and bridged by tri-dentate oxalate ligands to generate a one-dimensional infinite polymeric chain, [Mg(C_2_O_4_)(H_2_O)]_n_. Two neighboring *inversion-center-related* [Mg(C_2_O_4_)(H_2_O)]_n_ chains are further bridged by tetra-dentate oxalate ligands to complete the octahedral coordination sphere of Mg^2+^ and to form a ladder-like double chain with the composition [Mg_2_(C_2_O_4_)_3_(H_2_O)_2_]_n_^2n-^ (Fig. 2 b). Mg···Mg distances along the double chain are 5.846 (1) Å, slightly longer than those across the chain (5.390 (1) Å). The [Mg_2_(C_2_O_4_)_3_(H_2_O)_2_]_n_^2n-^ chains extend along the [100] direction and pack in two orientations in a herringbone pattern, as illustrated in Fig.2a. These chains are held together *via* medium-to-weak O—H···O hydrogen bonds existing between the coordinated H_2_O molecules and the O atoms from tri-dentate oxalate ligands (Table 1). Na^+^ cations are located in the void space between the chains to balance charge.

There is one crystallographically independent Na^+^ cation, which is coordinated to seven O atoms, forming an irregular coordination polyhedral geometry. The Na—O distances range from 2.2952 (10) to 2.8074 (10) Å, with an average of 2.510 Å, which is comparable to the value 2.46 Å computed from crystal radii for a 7-coordinated Na^+^ ion (Shannon, 1976) and the distances 2.409 (3)–2.606 (3) Å (average 2.505 Å, CN = 7) in NaLi_2_BO_3_ (Miessen & Hoppe, 1987). Bond valence sum (BVS) calculations using Brown's formula (Brown & Altermatt,1985) produced a BVS value of 1.15 for Na, in good agreement with its expected formal valence. The Mg atom also occupies one crystallographically distinct site. However, each Mg^2+^ is coordinated by six O atoms, five of which are from three oxalate ions and the other from one H_2_O molecule. The MgO_6_ octahedron is strongly distorted, with the 180° octahedral angles being 162.33 (4)–171.66 (3)°, and the 90° octahedral angles in the range 78.40 (3)–99.05 (4)°, the smallest angle being associated with the constrained Mg1—O4^i^—C2^i^ –C3^i^ –O5^i^ five-membered ring [Symmetry codes: (i) -1 + *x*, *y*, *z*]. The Mg—O distances of 2.0436 (9)–2.1429 (9) Å (average 2.078 Å) are very reasonable when compared with the ranges 2.057 (9)–2.080 (9) Å (average 2.065 Å) in Mg(NO_3_)_2_.6H_2_O, where octahedrally coordinated Mg^2+^ is also found (Schefer & Grube, 1995). The calculated BVS value for Mg is also reasonable, at 2.12. Of the two unique oxalate ions, the C1-based oxalate sits on an inversion center and the C2/C3-based one on a general position. Both oxalate ions are nearly planar, with a mean deviation of 0.0004 and 0.1418 Å, respectively, and the bond geometries of [C_2_O_4_]^2-^ are in accord with those observed in other oxalate compounds (Lethbridge *et al.*, 2003).

### Experimental

The title compound was synthesized by a two-step process. First, for the preparation of the precursor, Na_3_MgB_5_O_10_, a stoichiometric mixture of Na_2_CO_3_, MgO, and H_3_BO_3_ was heated at 873 K for two weeks with several intermediate re-mixings and the resulting product was identified to be the pure phase of Na_3_MgB_5_O_10_ based on the powder XRD analysis. Then, a 0.300 g (0.976 mmol) sample of Na_3_MgB_5_O_10_, 0.300 g (2.380 mmol) H_2_(C_2_O_4_).2H_2_O, and 3 ml H_2_O were sealed in an 15-ml Teflon-lined autoclave and subsequently heated at 453 K for one week, then cooled slowly to room temperature. The product consisted of colorless, prismatic crystals with the largest having dimensions of 0.6 × 0.6 × 1.2 mm^3^ in colorless mother liquor. The final pH of the reaction system was about 2.0. The crystals were isolated in about 70% yield (based on Mg) by washing the reaction product with deionized water and anhydrous ethanol followed by drying with anhydrous acetone. The powder XRD pattern of the ground crystals is in good agreement with that calculated from the single-crystal data, confirming that the pure phase of the title compound has been obtained. Although boron was not incorporated into the final structure, borate anions may serve as mineralizers to enhance the crystal growth.

### Refinement

H-atom positions were located in a difference Fourier map and all associated parameters were refined freely.

### Crystal data

**Table:**

Na_2_[Mg_2_(C_2_O_4_)_3_(H_2_O)_2_]	*F*(000) = 396
*M**_r_* = 394.70	*D*_x_ = 2.070 Mg m^−^^3^
Monoclinic, *P*2_1_/*c*	Mo *K*α radiation, λ = 0.71073 Å
Hall symbol: -P 2ybc	Cell parameters from 25 reflections
*a* = 5.8460 (12) Å	θ = 21.9–22.5°
*b* = 15.726 (3) Å	µ = 0.34 mm^−^^1^
*c* = 7.0190 (14) Å	*T* = 290 K
β = 101.11 (3)°	Prism, colorless
*V* = 633.2 (2) Å^3^	0.4 × 0.2 × 0.2 mm
*Z* = 2	

### Data collection

**Table:**

Rigaku AFC-7R diffractometer	2027 reflections with *I* > 2σ(*I*)
Radiation source: fine-focus sealed tube	*R*_int_ = 0.029
graphite	θ_max_ = 32.5°, θ_min_ = 2.6°
2θ–ω scans	*h* = 0→8
Absorption correction: ψ scan (Kopfmann & Huber, 1968)	*k* = 0→23
*T*_min_ = 0.912, *T*_max_ = 0.943	*l* = −10→10
2457 measured reflections	3 standard reflections every 150 reflections
2280 independent reflections	intensity decay: 1.2%

### Refinement

**Table:**

Refinement on *F*^2^	Secondary atom site location: difference Fourier map
Least-squares matrix: full	Hydrogen site location: difference Fourier map
*R*[*F*^2^ > 2σ(*F*^2^)] = 0.033	All H-atom parameters refined
*wR*(*F*^2^) = 0.095	*w* = 1/[σ^2^(*F*_o_^2^) + (0.0581*P*)^2^ + 0.0731*P*] where *P* = (*F*_o_^2^ + 2*F*_c_^2^)/3
*S* = 1.11	(Δ/σ)_max_ = 0.001
2280 reflections	Δρ_max_ = 0.54 e Å^−^^3^
118 parameters	Δρ_min_ = −0.56 e Å^−^^3^
0 restraints	Extinction correction: *SHELXL97* (Sheldrick, 2008), Fc^*^=kFc[1+0.001xFc^2^λ^3^/sin(2θ)]^-1/4^
Primary atom site location: structure-invariant direct methods	Extinction coefficient: 0.310 (14)

### Special details

**Table:**

Geometry. All e.s.d.'s (except the e.s.d. in the dihedral angle between two l.s. planes) are estimated using the full covariance matrix. The cell e.s.d.'s are taken into account individually in the estimation of e.s.d.'s in distances, angles and torsion angles; correlations between e.s.d.'s in cell parameters are only used when they are defined by crystal symmetry. An approximate (isotropic) treatment of cell e.s.d.'s is used for estimating e.s.d.'s involving l.s. planes.
Refinement. Refinement of *F*^2^ against ALL reflections. The weighted *R*-factor *wR* and goodness of fit *S* are based on *F*^2^, conventional *R*-factors *R* are based on *F*, with *F* set to zero for negative *F*^2^. The threshold expression of *F*^2^ > σ(*F*^2^) is used only for calculating *R*-factors(gt) *etc*. and is not relevant to the choice of reflections for refinement. *R*-factors based on *F*^2^ are statistically about twice as large as those based on *F*, and *R*- factors based on ALL data will be even larger.

### Fractional atomic coordinates and isotropic or equivalent isotropic displacement parameters (Å^2^)

**Table:**

	*x*	*y*	*z*	*U*_iso_*/*U*_eq_	
Na1	0.46789 (7)	0.81852 (3)	0.30526 (7)	0.01952 (13)	
Mg1	0.27308 (6)	0.60144 (2)	0.21580 (5)	0.01297 (12)	
C1	0.49584 (17)	0.46394 (6)	0.42523 (14)	0.01492 (18)	
O1	0.38596 (15)	0.47765 (5)	0.25634 (11)	0.01885 (17)	
O2	0.60143 (15)	0.39673 (5)	0.48571 (11)	0.02059 (18)	
C2	0.79984 (15)	0.64930 (6)	0.21459 (13)	0.01346 (18)	
O3	0.58217 (13)	0.65770 (5)	0.17720 (13)	0.02067 (17)	
O4	0.91349 (13)	0.58130 (5)	0.22737 (12)	0.01867 (17)	
C3	0.94672 (16)	0.73184 (6)	0.24642 (14)	0.01425 (18)	
O5	1.15751 (12)	0.72335 (5)	0.23254 (12)	0.01735 (17)	
O6	0.85117 (14)	0.79871 (5)	0.28409 (15)	0.0255 (2)	
O7	0.18307 (14)	0.58554 (5)	−0.08136 (12)	0.01742 (16)	
H7A	0.174 (4)	0.5388 (16)	−0.124 (4)	0.058 (7)*	
H7B	0.065 (5)	0.6120 (18)	−0.141 (4)	0.077 (9)*	

### Atomic displacement parameters (Å^2^)

**Table:**

	*U*^11^	*U*^22^	*U*^33^	*U*^12^	*U*^13^	*U*^23^
Na1	0.0152 (2)	0.0208 (2)	0.0230 (2)	−0.00005 (15)	0.00461 (16)	0.00233 (16)
Mg1	0.01111 (17)	0.01020 (17)	0.01744 (18)	0.00096 (10)	0.00233 (12)	0.00035 (10)
C1	0.0171 (4)	0.0111 (4)	0.0166 (4)	0.0015 (3)	0.0031 (3)	−0.0012 (3)
O1	0.0258 (4)	0.0130 (3)	0.0161 (3)	0.0031 (3)	−0.0001 (3)	−0.0011 (2)
O2	0.0289 (4)	0.0134 (3)	0.0179 (3)	0.0080 (3)	0.0005 (3)	−0.0014 (2)
C2	0.0104 (4)	0.0140 (4)	0.0165 (4)	−0.0015 (3)	0.0039 (3)	−0.0008 (3)
O3	0.0100 (3)	0.0223 (4)	0.0300 (4)	−0.0018 (3)	0.0045 (3)	0.0009 (3)
O4	0.0147 (3)	0.0120 (3)	0.0297 (4)	−0.0007 (2)	0.0054 (3)	−0.0008 (3)
C3	0.0105 (4)	0.0120 (4)	0.0200 (4)	0.0002 (3)	0.0022 (3)	−0.0009 (3)
O5	0.0102 (3)	0.0113 (3)	0.0310 (4)	0.0000 (2)	0.0051 (3)	−0.0008 (3)
O6	0.0150 (3)	0.0144 (3)	0.0470 (5)	0.0026 (3)	0.0062 (3)	−0.0083 (3)
O7	0.0176 (3)	0.0148 (3)	0.0191 (3)	0.0019 (3)	0.0017 (3)	−0.0011 (2)

### Geometric parameters (Å, °)

**Table:**

Na1—O6	2.2952 (10)	Mg1—O4^i^	2.1429 (9)
Na1—O5^i^	2.3315 (9)	C1—O1	1.2533 (12)
Na1—O2^ii^	2.3514 (10)	C1—O2	1.2559 (11)
Na1—O7^iii^	2.4886 (10)	C1—C1^iv^	1.5399 (19)
Na1—O3^iii^	2.5941 (12)	C2—O4	1.2531 (12)
Na1—O1^ii^	2.7059 (10)	C2—O3	1.2557 (11)
Na1—O3	2.8074 (10)	C2—C3	1.5485 (13)
Mg1—O5^i^	2.0436 (9)	C3—O6	1.2428 (12)
Mg1—O1	2.058 (1)	C3—O5	1.2618 (11)
Mg1—O7	2.0656 (10)	O7—H7A	0.79 (3)
Mg1—O3	2.0761 (9)	O7—H7B	0.85 (3)
Mg1—O2^iv^	2.0823 (10)		
			
O6—Na1—O5^i^	128.83 (4)	O5^i^—Mg1—O2^iv^	89.17 (3)
O6—Na1—O2^ii^	91.25 (4)	O1—Mg1—O2^iv^	80.36 (3)
O5^i^—Na1—O2^ii^	98.57 (4)	O7—Mg1—O2^iv^	171.66 (3)
O6—Na1—O7^iii^	145.56 (3)	O3—Mg1—O2^iv^	88.78 (5)
O5^i^—Na1—O7^iii^	85.34 (3)	O5^i^—Mg1—O4^i^	78.40 (3)
O2^ii^—Na1—O7^iii^	86.94 (4)	O1—Mg1—O4^i^	98.38 (4)
O6—Na1—O3^iii^	91.11 (4)	O7—Mg1—O4^i^	87.68 (5)
O5^i^—Na1—O3^iii^	110.58 (4)	O3—Mg1—O4^i^	162.33 (4)
O2^ii^—Na1—O3^iii^	140.04 (3)	O2^iv^—Mg1—O4^i^	97.00 (5)
O7^iii^—Na1—O3^iii^	69.46 (3)	O1—C1—O2	126.40 (9)
O6—Na1—O1^ii^	76.88 (3)	O1—C1—C1^iv^	117.46 (10)
O5^i^—Na1—O1^ii^	144.59 (3)	O2—C1—C1^iv^	116.14 (11)
O2^ii^—Na1—O1^ii^	52.00 (3)	C1—O1—Mg1	112.70 (6)
O7^iii^—Na1—O1^ii^	75.03 (3)	C1—O2—Mg1^iv^	112.55 (6)
O3^iii^—Na1—O1^ii^	89.97 (3)	O4—C2—O3	127.35 (9)
O6—Na1—O3	63.99 (3)	O4—C2—C3	115.69 (8)
O5^i^—Na1—O3	64.84 (3)	O3—C2—C3	116.96 (8)
O2^ii^—Na1—O3	101.83 (3)	C2—O3—Mg1	143.16 (7)
O7^iii^—Na1—O3	149.74 (3)	C2—O4—Mg1^v^	112.43 (6)
O3^iii^—Na1—O3	114.90 (3)	O6—C3—O5	126.27 (9)
O1^ii^—Na1—O3	132.84 (3)	O6—C3—C2	118.71 (8)
O5^i^—Mg1—O1	168.63 (4)	O5—C3—C2	115.01 (8)
O5^i^—Mg1—O7	98.56 (4)	C3—O5—Mg1^v^	116.25 (6)
O1—Mg1—O7	92.16 (3)	Mg1—O7—H7A	118.6 (19)
O5^i^—Mg1—O3	85.03 (3)	Mg1—O7—H7B	117.6 (19)
O1—Mg1—O3	99.05 (4)	H7A—O7—H7B	106 (2)
O7—Mg1—O3	88.77 (4)		

Symmetry codes: (i) *x*−1, *y*, *z*; (ii) −*x*+1, *y*+1/2, −*z*+1/2; (iii) *x*, −*y*+3/2, *z*+1/2; (iv) −*x*+1, −*y*+1, −*z*+1; (v) *x*+1, *y*, *z*.

### Hydrogen-bond geometry (Å, °)

**Table:**

*D*—H···*A*	*D*—H	H···*A*	*D*···*A*	*D*—H···*A*
O7—H7a···O4^vi^	0.78 (3)	2.06 (3)	2.8335 (13)	170 (2)
O7—H7b···O6^vii^	0.84 (3)	1.89 (3)	2.6952 (13)	162 (3)

Symmetry codes: (vi) −*x*+1, −*y*+1, −*z*; (vii) *x*−1, −*y*+3/2, *z*−1/2.
